# Stories about Dogs

**Published:** 1887-07-23

**Authors:** F. O. Morris

**Affiliations:** Rector of Nunburnholme, Yorkshire; author of "A History of British Birds." etc.


### Incidents of Animal Life.

Collated by the Rev. F. O. MORRIS, B.A.,
Rector of Nunburnholme, Yorkshire; author of "A History of British Birds," etc.

"He prayeth best, who loveth best
All things both great and small;
For the dear God who loveth us,
He made and loveth all."—COLERIDGE.

### Stories about Dogs.

Mr. H. Hawkes, a farmer, residing at Malling, in Kent, was late one evening at Maidstone market. On returning at night with his dog, which was usually at his heels, he took his way over Snodlane brook. The snow fell so fast that he lost his way, and being exhausted, fell down, and turning upon his back, was soon overpowered with either sleep or cold. His faithful dependent first scratched away the snow, so as to throw up a sort of protecting wall around his helpless master, then mounted upon the exposed body, rolled round, and lay down upon his master's bosom, for which his shaggy coat proved a most seasonable covering and eventful protection during the dreadful severity of the night, the snow falling all the time. The following morning, a person who was out with his gun, perceiving an appearance rather uncommon, ventured to approach the spot; upon his coming up, the dog got off the body, and, after repeatedly shaking himself to get disentangled from the accumulated snow, encouraged the sportsman, by actions of the most significant nature, to come near the side of his master. Upon wiping away the icy incrustation from the face, the countenance was immediately recollected, but the frame appeared lifeless. Assistance was procured to convey the body to the first house upon the skirts of the village, when, a pulsation being observed, every possible means was instantly adopted to promote his recovery. After a short time the farmer was sufficiently restored to tell the story we have related, and in gratitude for his miraculous escape, ordered a silver collar to be made for his friendly protector, as a perpetual remembrance of the transaction. A medical gentleman in the neighbourhood, hearing of the circumstance, and finding it so well authenticated, immediately made an offer of ten guineas for the dog, which the grateful farmer refused, exultingly adding, "So long as I have a crust to my meat, or a bone to my bread, I will divide it with the faithful friend who has preserved my life"; and this he did in a perfect conviction that the warmth of the dog, in covering the most vital part, had continued the circulation, and prevented a total stagnation of the blood by the frigidity of the elements.

---

During Landseer's four years' illness Tiney never left his side. In the garden, on very fine days, the faithful dog would sit coiled up for hours at his master's feet; and shortly before his end, Landseer, embracing his pet, exclaimed, "My dear little white dog, nobody can love me half as much as thou dost."

---

"*Greyfriars Bobby.*"—A poor labouring man died, and was buried in the old Greyfriars Churchyard, Edinburgh. It was a plain, undistinguished grave, but there, with a few intervals of absence, by day and night, his little terrier dog was seen to remain. How it was supported none could tell, but after a time one who resided near the churchyard used to give the poor faithful animal its food. When the dog-tax was imposed the collector came upon Bobby's new patron for the tax. He explained that he was not the owner of the dog, whose master lay buried in the churchyard. The matter came before the city magistrates on appeal. Inquiries were made, and it was found that Bobby had, with touching fidelity, clung to the memory and to the grave of his master. The Lord Provost and the magistrates of Edinburgh obtained for Bobby exemption from the tax, and presented the faithful creature with a collar with a suitable inscription. He continued to live in the churchyard till he died. The story had long before become famous, and the generous, kind-hearted Baroness Burdett-Coutts has since erected a monument to Bobby's memory, in the form of a drinking fountain surmounted by a sculptured effigy of the dog.

---

You will often see in the country a little dog sitting beside a small heap of clothes, with perhaps a tin can, and a staff, and a basket. Don't go near him, don't disturb him; he is rather spiteful now, but for that very reason deserves respect; for he is minding his master's jacket and other properties, while he is at his work in the field. Not long ago there was an account in the papers of a drover who left his dog to mind his jacket while he went across a railway to look after some cattle. In crossing the railway the poor man was struck down by a train and killed. The dog never left its charge, but died in guarding its dead master's jacket.

